# ImmunoPET imaging of amyloid-beta in a rat model of Alzheimer’s disease with a bispecific, brain-penetrating fusion protein

**DOI:** 10.1186/s40035-022-00324-y

**Published:** 2022-12-26

**Authors:** Gillian Bonvicini, Stina Syvänen, Ken G. Andersson, Merja Haaparanta-Solin, Francisco López-Picón, Dag Sehlin

**Affiliations:** 1 Department of Public Health and Caring Sciences, Uppsala University, 751 85 Uppsala, Sweden; 2 BioArctic AB, 112 51 Stockholm, Sweden; 3 Preclinical Imaging Laboratory, Turku PET Centre, University of Turku, 20520 Turku, Finland; 4 MediCity Research Laboratory, University of Turku, 20520 Turku, Finland

**Keywords:** ImmunoPET, Transferrin receptor, Alzheimer’s disease, Amyloid-beta

## Abstract

**Background:**

Hijacking the transferrin receptor (TfR) is an effective strategy to transport amyloid-beta (Aβ) immuno-positron emission tomography (immunoPET) ligands across the blood–brain barrier (BBB). Such ligands are more sensitive and specific than small-molecule ligands at detecting Aβ pathology in mouse models of Alzheimer’s disease (AD). This study aimed to determine if this strategy would be as sensitive in rats and to assess how TfR affinity affects BBB transport of bispecific immunoPET radioligands.

**Methods:**

Two affinity variants of the rat TfR antibody, OX26, were chemically conjugated to a F(ab′)_2_ fragment of the anti-Aβ antibody, bapineuzumab (Bapi), to generate two bispecific fusion proteins: OX26_5_-F(ab′)_2_-Bapi and OX26_76_-F(ab′)_2_-Bapi. Pharmacokinetic analyses were performed 4 h and 70 h post-injection of radioiodinated fusion proteins in wild-type (WT) rats. [^124^I]I-OX26_5_-F(ab′)_2_-Bapi was administered to TgF344-AD and WT rats for in vivo PET imaging. Ex vivo distribution of injected [^124^I]I-OX26_5_-F(ab′)_2_-Bapi and Aβ pathology were assessed.

**Results:**

More [^125^I]I-OX26_5_-F(ab′)_2_-Bapi was taken up into the brain 4 h post-administration than [^124^I]I-OX26_76_-F(ab′)_2_-Bapi. [^124^I]I-OX26_5_-F(ab′)_2_-Bapi PET visualized Aβ pathology with significantly higher signals in the TgF344-AD rats than in the WT littermates without Aβ pathology. The PET signals significantly correlated with Aβ levels in AD animals.

**Conclusion:**

Affinity to TfR affects how efficiently a TfR-targeting bispecific fusion protein will cross the BBB, such that the higher-affinity bispecific fusion protein crossed the BBB more efficiently. Furthermore, bispecific immunoPET imaging of brain Aβ pathology using TfR-mediated transport provides good imaging contrast between TgF344-AD and WT rats, suggesting that this immunoPET strategy has the potential to be translated to higher species.

**Supplementary Information:**

The online version contains supplementary material available at 10.1186/s40035-022-00324-y.

## Background

Positron emission tomography (PET) is a useful diagnostic tool for neurodegenerative diseases to visualize and quantify aspects of pathology inside the brain in vivo. For example, visualization of amyloid-beta (Aβ) plaques in the brains of Alzheimer’s disease (AD) patients with [^11^C]Pittsburgh Compound B ([^11^C]PiB) or other fluorine-18 (^18^F)-labelled analogues increases the diagnostic confidence of AD [1]. However, these small-molecule tracers do not bind to soluble Aβ aggregates that are assumed to be the toxic species underlying early AD progression [2, 3] or to diffuse Aβ plaques such as those found in AD patients with the Arctic APP mutation [4]. Small-molecule tracers also perform poorly in discriminating between different proteins with similar fibrillary structures (e.g., Aβ vs alpha-synuclein, a protein which aggregates in Parkinson’s disease) [5].

Antibody-based tracers are highly specific to their targets, and were increasingly used for peripheral PET in the oncology field during the last decade [6, 7]. However, one of the main reasons that hinder the use of immunoPET radioligands for central nervous system (CNS) targets is their limited blood–brain barrier (BBB) passage. Only less than 0.05% of the injected antibody dose passes into the brain 2 h after injection, making it difficult to achieve adequate signal-to-noise ratios for in vivo imaging [8–11].

Antibodies can be engineered to utilize endogenous active transport systems at the BBB to enhance brain uptake, such as transferrin receptor (TfR)-mediated transcytosis [12–14]. Targeting the TfR is an effective strategy for increasing brain delivery of therapeutic antibodies and peptides in mice [10, 15–19], rats [20–23], non-human primates [24–26], and human subjects in some clinical trials [27]. This strategy has also proven successful for developing bispecific antibody-based radioligands for Aβ PET in AD mouse models [7, 9–11, 28–33].

The affinity of bispecific antibodies to TfR influences their BBB-crossing efficacy, and there is an optimal TfR affinity window at therapeutic doses [16, 18, 22, 24, 34–37]. Antibodies with too high affinity are not released on the abluminal side of brain endothelial cells, while antibodies with too low affinity bind to TfR insufficiently for transcytosis and remain in the blood [16, 18, 22, 24]. This relationship is potentially dose-related. At tracer doses, increasing the affinity directly correlates with higher brain uptake [16].

Bispecific antibody-based radioligands targeting both TfR and Aβ have been shown to detect Aβ pathology and discriminate between AD and WT mice at an earlier disease stage than [^11^C]PiB [28, 31, 32]. Moreover, a bispecific antibody-based radioligand is more sensitive than [^11^C]PiB at detecting reductions in Aβ levels in two AD mouse models after treatment with an Aβ-reducing BACE-1 inhibitor [7]. These studies indicate that the bispecific immunoPET radioligands can image more subtle changes in Aβ pathology than small-molecule tracers.

Given this background, the primary aim of this study was to evaluate if immunoPET imaging of Aβ aggregates is equally sensitive in rats as in mice. The second aim was to assess the role of TfR affinity in transport of immunoPET radioligands across the BBB, using two bispecific fusion proteins with different TfR-affinity, OX26_5_-F(ab′)_2_-Bapi and OX26_76_-F(ab′)_2_-Bapi.

## Materials and methods

### Recombinant protein expression and purification

Two variants of the mouse anti-rat TfR (rTfR) antibody, OX26, were produced: OX26_5_ and OX26_76_ [22, 38]. OX26_5_ is the WT OX26 antibody and OX26_76_ has a single alanine mutation in the complementarity determining region 1 on the heavy chain variable region. Both OX26 variants were expressed as mouse IgG2c molecules. The extracellular domain of rTfR (L101 to F761) was also produced with flag and 10xHis tags attached to the C-terminus.

Expression vectors pcDNA3.4 were synthesized by GeneArt Elements (Invitrogen, Carlsbad, CA) and were transfected into the Expi293 Expression System following the manufacturer’s instructions (Life Technologies, Carlsbad, CA). Plasmid DNA (100 µg total; 1:1 ratio of heavy to light chain for antibodies) and ExpiFectamine 293 reagent in Opti-MEM® I medium were added to the Expi293F cells. Cells were incubated at 37 °C, 120 RPM, with 70% humidity and 8% CO_2_ for 20 h before ExpiFectamine 293 Transfection enhancer 1 and 2 were added. Four days later, cell supernatant was harvested for purification.

OX26 variants were purified on a HiTrap Protein G column (Cytiva, Uppsala, Sweden) with an ÄKTA Purifier system (Cytiva) and eluted with an increasing gradient of 0.7% acetic acid (HAc). rTfR was purified on a HisTrap Excel immobilized metal ion affinity chromatography column (Cytiva) with a binding buffer containing 20 mM Tris and 200 mM NaCl and eluted in the binding buffer with 500 mM imidazole. Following elution, all three proteins underwent buffer exchange to phosphate buffered saline (PBS) on a HiPrep 26/10 desalting column (Cytiva).

### Biacore analysis of the OX26 affinity to rTfR

With the Biacore 8 K (Cytiva), anti-mouse IgG from the Mouse Antibody Capture Kit, type 2 (Cytiva) was immobilized on flow cells 1 and 2 of a CM5 chip (Cytiva) following kit instructions. Ten micrograms of OX26_5_, OX26_76_, or a commercial control OX26 (LS-C43741, LSBio, Seattle, WA) were captured on flow cell 2. A 1-min regeneration step with 10 nM glycine-HCl (pH 1.7) on flow cell 1 succeeded the capture step to ensure no antibody was captured on the reference flow cell. A single cycle kinetics assay with 5 concentration steps from 3.2 to 2000 nM of rTfR for 2 min each was run over both flow cells, followed by 1 h of dissociation. All dilutions were done in HBS-EP + running buffer (0.01 M HEPES, 0.15 M NaCl, 3 mM EDTA and 0.05% *v*/*v* Surfactant P20, Cytiva) supplemented with 0.1% *w*/*v* bovine serum albumin (BSA). Data were analysed with Biacore Insight Evaluation 3.0.12.15655 (Cytiva) and was fitted with a 1:1 binding model.

### F(ab′)_2_ fragmentation of bapineuzumab (Bapi)

Bapineuzumab (Bapi; Absolute Antibody, Oxford, UK), a humanized monoclonal antibody binding Aβ based on the murine antibody 3D6 and former clinical therapeutic candidate [39], was enzymatically cleaved into F(ab′)_2_ fragments (F(ab′)_2_-Bapi) with the cysteine protease IdeS in FragIT columns (Genovis AB, Lund, Sweden). IdeS produces a homogenous preparation of F(ab′)_2_ fragments by cleaving human IgG at a specific site below the hinge region. F(ab′)_2_ fragments were purified from Fc fragments and uncleaved antibodies with CaptureSelect Fc (multi-species) Affinity Resin (ThermoFisher Scientific, Stockholm, Sweden). The F(ab′)_2_ purity was assessed with SDS-PAGE under non-reducing conditions.

### Chemical conjugation and purification of bispecific fusion proteins

Trans-cyclooctene (TCO)-functionalized OX26_5_ or OX26_76_ was chemically conjugated to tetrazine-functionalized F(ab′)_2_-Bapi via an inverse-electron-demand Diels–Alder (IEDDA) reaction to produce two bispecific fusion proteins: OX26_5_-F(ab′)_2_-Bapi and OX26_76_-F(ab′)_2_-Bapi.

To prepare for chemical conjugation, OX26_5_ or OX26_76_ (2 mg/ml) was incubated with a 20-fold molar excess of axial TCO-NHS (Conju-Probe, LLC, San Diego, CA) and F(ab′)_2_-Bapi (4 mg/ml) with a 7-fold molar excess of Tetrazine-PEG5-NHS (Sigma-Aldrich, Stockholm, Sweden) in PBS with 30 mM carbonate buffer (pH 9.6) for 2.5 h, with shaking in darkness at room temperature (RT). After incubation, the buffer was exchanged to PBS with Zeba spin desalting columns 7 K MWCO to remove any free TCO-NHS or Tetrazine-PEG5-NHS. Modified OX26 affinity variants were incubated with 1.5-fold molar excess of modified F(ab′)_2_-Bapi, with shaking in darkness at RT. After 30 min, a 200-fold molar excess of methyltetrazine-amine HCl salt (Click Chemistry Tools, Scottsdale, AZ) was added to stop any unreacted TCOs from conjugating to unreacted tetrazine on F(ab′)_2_-Bapi and forming large multi-antibody complexes. Reactions were incubated for another 30 min in darkness before free tetrazine was removed with Zeba spin desalting columns 7k MWCO. Proteins were separated via gel filtration chromatography on a HiLoad® 26/600 Superdex® 200 prep grade column (Cytiva) using an ÄKTA Purifier system. Components of each fraction were assessed with SDS-PAGE. Approximately 2 µg of protein was mixed with LDL Sample Buffer (ThermoFisher Scientific), loaded onto a NuPAGE 3%–8% Tris-Acetate gel (ThermoFisher Scientific) and run at 150 V for 1 h with NuPAGE Tris-Acetate SDS Running Buffer (ThermoFisher Scientific). The gel was washed in water, fixed in 50% methanol and 7% HAc solution, and stained with GelCode Blue Stain Reagent (ThermoFisher Scientific).

### Radiochemistry

OX26_5_-F(ab′)_2_-Bapi and OX26_76_-F(ab′)_2_-Bapi were radiolabelled with iodine-124 (^124^I) or iodine-125 (^125^I) by the direct iodination Chloramine-T method (Table 1) [40].

**Table 1:** Radiochemical reaction yield and specific activity of radioligands

Radioligand	Reaction yield (%)	Molar activity (MBq/nmol)
[^124^I]I-OX26_5_F(ab′)_2_Bapi	57.0 ± 6.9	60.0 ± 24.2
[^125^I]I-OX26_5_F(ab′)_2_Bapi	21.6 ± 11.2	26.5 ± 26.6
[^124^I]I-OX26_76_F(ab′)_2_Bapi	58.5 ± 3.1	209.1 ± 5.9

For ^124^I-labelling, 31.5 ± 9.9 MBq of ^124^I stock solution (Advanced Centre Oncology Macerata, Montecosaro, Italy) was pre-incubated with 33.3% *v*/*v* sodium iodine (NaI, 50 µM) for 15 min, and then neutralized with 0.5% HAc and 11.1% *v*/*v* 10xPBS. Then, 92.9 ± 57.2 µg of OX26_5_-F(ab′)_2_-Bapi or 37.0 ± 0 µg of OX26_76_-F(ab′)_2_-Bapi and Chloramine-T (final concentration 0.1 mg/ml, Sigma-Aldrich) were added to the pre-incubated ^124^I solution. The reaction was quenched after 120 s by adding sodium metabisulfite (final concentration 0.2 mg/ml, Sigma-Aldrich).

For ^125^I-labelling, either 60 µg of OX26_5_-F(ab′)_2_-Bapi or 23.4 µg of OX26_5_-F(ab′)_2_-Bapi modified with Bolton-Hunter reagent, as described previously [28], was mixed with 11.1 ± 1.1 MBq of ^125^I stock solution (PerkinElmer Inc., Waltham, MA), Chloramine-T (final concentration 40 µg/ml) and PBS to a final volume of 110 µl. After incubating 90 s at room temperature, the reaction was stopped with sodium metabisulfite (final concentration 74 µg/ml).

Radiolabelled products were diluted to 500 µl with PBS, purified of free iodine with a disposable NAP-5 size exclusion column (Cytiva) and eluted in 1 ml of PBS. Radiolabelling was performed no more than 2 h prior to each in vivo study.

### Quality control of radiolabelled bispecific fusion proteins

Sandwich ELISA was performed to determine the concentrations of bispecific fusion proteins after radiolabelling using plates coated with 2 nM anti-mouse-IgG (#AI-2000, Vector Laboratories Inc., Newark, CA). Indirect ELISA was performed to assess the potential effects of conjugation and radiolabelling on the binding of OX26_5_-F(ab′)_2_-Bapi or OX26_76_-F(ab′)_2_-Bapi to rTfR and Aβ, using plates coated with 13.3 nM of rTfR or 50 nM of Aβ (Innovagen, Lund, Sweden), respectively.

ELISA assays were performed in 96-well half-area plates (Corning Inc., New York, NY). Plates were coated with respective proteins diluted in PBS overnight at 4 °C and then blocked with 1% BSA in PBS for 1 h. All further dilutions were made in ELISA incubation buffer (PBS with 0.1% BSA, 0.05% Tween and 0.15% Kathon). Control antibodies (OX26_5_, OX26_76_ and Bapi) and the bispecific fusion proteins before and after radiolabelling were serially diluted from 50 nM to 3.2 pM, incubated overnight at 4 °C, washed and then detected with horseradish peroxidase (HRP)-coupled goat anti-mouse IgG-F(ab′)_2_ (1:2000, #115-035-006, Jackson ImmunoResearch Laboratories, West Grove, PA) or goat anti-human IgG-F(ab′)_2_ (1:2000, #109-036-006, Jackson ImmunoResearch Laboratories). Signals were developed with K blue aqueous TMB substrate (Neogen Corp., Lexington, KY), halted with 1 M H_2_SO_4_ and read with a spectrophotometer at 450 nm. The EC50 values were calculated from agonist concentration vs response curves with variable slope (four parameters) where the bottom was constrained to 0 in GraphPad Prism.

### Animals

Animals were housed with *ad libitum* access to food and water in an approved animal facility at Uppsala University with controlled temperature and humidity. All procedures in this study were approved by the Uppsala County Animal Ethics board (5.8.18–20401/2020) following the rules and regulations of the Swedish Animal Welfare Agency and in compliance with the European Communities Council Directive of 22 September 2010 (2010/63/EU).

WT Fischer 344 rats (Janvier Labs, Le Genest-Saint Isle, France) were used in the pharmacokinetic studies, and TgF344-AD rats and WT littermates for PET scans. The TgF344-AD rats express human APP with the Swedish mutation (*AβPP KM670/671NL*) and human PSEN1 with exon 9 deletion (*PS1-ΔE9*). They begin displaying age-dependent Aβ plaque pathology at 6 months [41, 42] and do not show any sex differences in Aβ pathology load [41, 43].

### Pharmacokinetic study

Three-month-old male WT rats were lightly sedated with isoflurane (Isoflurane Baxter®, Baxter Medical AB, Kista, Sweden) and injected in the tail vein with either [^125^I]I-OX26_5_-F(ab′)_2_-Bapi or [^124^I]I-OX26_76_-F(ab′)_2_-Bapi (Table 2). The rats were anaesthetized with isoflurane 4 h or 70 h post-administration and a terminal blood sample was taken from the heart. The rats were then euthanized by transcardial perfusion with 130 ml of 0.9% NaCl in 8 min to clear the brain and organs of blood. The brain was isolated and dissected into olfactory bulbs, right hemisphere, left cortex, left midbrain and left cerebellum. Brain samples were immediately frozen on dry ice, except for left cortices used for capillary depletion. Lung, heart, liver, pancreas, spleen, kidney, femoral bone, femoral bone marrow, and skull were isolated and a urine sample was collected. Radioactivity from samples was measured with a γ-counter (2480 Wizard™, PerkinElmer Inc.). Concentrations of the bispecific fusion proteins were expressed as standardized uptake values (SUV) to account for variation in weight between animals.

**Table 2:** Number of rats (male/female), injected radioactivity, and dose for the pharmacokinetic (PK) and the PET studies

Radioligand	PK study		PET study		Injected radioactivity (MBq)	Injected dose (mg/kg)
	WT		WT	TgF344-AD		
	4 h	70 h	3 days	3 days		
[^124^I]I-OX26_5_F(ab′)_2_Bapi	―	―	2/2	4/1	13.0 ± 6.1	0.12 ± 0.08
[^125^I]I-OX26_5_F(ab′)_2_Bapi	2/0	2/0	―	―	0.9 ± 0.1	0.04 ± 0.02
[^124^I]I-OX26_76_F(ab′)_2_Bapi	2/0	3/0	―	―	6.1 ± 1.3	0.04 ± 0.01

#### Blood pharmacokinetics

Blood samples (8 µl) were obtained from the tail vein at 1 h, 4 h, 24 h, and 48 h post-administration. Terminal blood was collected and plasma was separated from the blood cell pellet by centrifugation. Radioactivity was measured with a γ-counter to calculate blood concentration and the percent of free antibody in plasma.

Whole blood half-life was estimated with a non-linear regression two-phase decay model. The plateau was constrained to zero. Y0 was fixed to 14.3 based on the assumption that 100% of the injected dose enters the blood immediately after injection and the average rat blood volume is 7% of their body weight [44]. The fusion protein exposure, quantified as area under the curve (AUC), was calculated from the SUV blood curves.

#### Capillary depletion

Capillary depletion was performed on perfused left cortices of rats euthanized 4 h post-injection of [^125^I]I-OX26_5_-F(ab′)_2_-Bapi or [^124^I]I-OX26_76_-F(ab′)_2_-Bapi. Immediately after transcardial perfusion, cortices were isolated, weighed and homogenized in 2 ml cold physiological buffer (10 mM HEPES, 141 mM NaCl, 4 mM KCl, 2.8 mM CaCl_2_, 1 mM MgSO_4_, 1 mM NaH_2_PO_4_, 10 mM *D*-glucose, pH 7.4) with 8 strokes in an ice-cold Dounce homogeniser. Then 4 ml of 30% Ficoll 400 (Sigma-Aldrich) was added, followed by an additional 2 strokes. The homogenate was centrifuged at 5200 *g* for 20 min at 4 °C resulting in two fractions: a capillary-enriched pellet and a parenchymal supernatant. Measured radioactivity for each fraction was normalized to the total activity of the homogenate.

### PET/computed tomography (CT) imaging

Fourteen-month-old male and female TgF344-AD rats and WT littermates were lightly sedated and injected with [^124^I]I-OX26_5_-F(ab′)_2_-Bapi in the tail vein (Table 2). The day before injection, animals were given water containing 0.5% NaI to reduce ^124^I-uptake in the thyroid. After injection, the concentration of NaI was reduced to 0.2% until scanning.

Three days post-injection, rats underwent PET and CT scans. The animals were anesthetized with 5% sevoflurane in 50% medical oxygen and 50% air, and placed on the gantry of a nanoScan® PET/MRI 3T system (Mediso Medical Imaging Systems, Budapest, Hungary) in a prone position for a 60–120-min PET scan (Field of view = 9.8 cm). A 5-min CT scan was taken with a nanoScan® SPECT/CT system (Mediso Medical Imaging Systems) which is compatible with PET/magnetic resonance image (MRI) and allows for correct image co-registration.

PET data were reconstructed using a Tera-TomoTM 3D algorithm (Mediso Medical Imaging Systems) with 4 iterations and 6 subsets. CT data were reconstructed using Filter Back Projection. Further PET and CT image processing was performed with Amide 1.0.4 [45]. CT and PET scans were manually aligned to a T2-weighted MRI-based rat brain atlas [46]. The following regions of interest were outlined in the MRI: caudate putamen, hippocampi, parietal cortex, occipital cortex, cerebellum and olfactory bulbs. PET data were quantified as mean radioactivity concentrations during the scan expressed as SUV.

### Ex vivo analysis of TgF344-AD rats

Immediately after the CT scan, animals were euthanized and dissected as described above, except that brains were dissected into olfactory bulbs, right hemisphere, left cortex (left front half of the cortex), left hippocampus and left cerebellum. Brain samples were immediately frozen and the radioactivity in the collected tissues, blood and urine samples was measured with a γ-counter. Bispecific fusion protein concentrations were expressed as SUV_ex vivo_ to account for weight variations between animals.

#### Ex vivo autoradiography

Frozen hemispheres from PET/CT-scanned rats were cryosectioned (CM1850, Leica Biosystems, Nussloch, Germany) into 20-µm sagittal sections. Two sections from each animal were immediately exposed to a phosphor imaging plate (MS, MultiSensitive, PerkinElmer Inc.) for 7 days. The plates were scanned in a Typhoon phosphor imager (Cytiva) at 600 dots per inch. The resulting digital images were converted to a false colour scale (Royal) in ImageJ.

#### Aβ immunofluorescence

Sagittal brain slices were fixed in 4% paraformaldehyde and washed in PBS. Antigen retrieval was performed by boiling sections in 25 mM citrate buffer (pH 7.3) for 2 min and leaving them to cool to RT for 40 min. The sections were treated with 70% formic acid (FA) for 10 min, rinsed in milliQ water, washed in PBS and permeabilized in 0.4% Triton in PBS for 5 min. Primary antibody was added to the sections and incubated overnight at 4 °C with slow shaking. The next day, sections were washed in PBS. Secondary antibody was added and incubated for 1 h with shaking at RT, followed by three washes, before mounting with Vectashield Hard Set Mounting medium with DAPI (BioNordika, Solna, Sweden). Fluorescence images were acquired with a Zeiss Observer Z1 microscope (Carl Zeiss Imaging GmBH, Jena, Germany) and processed using ZEN software.

Aβ_42_ immunofluorescence was performed on sagittal brain slices from PET animals. The primary antibody was rabbit anti-human Aβ_42_ (#700,254, Invitrogen) diluted to 1 µg/ml in 0.1% Tween-20 in PBS. The secondary antibody was Alexa Fluor 488 goat anti-rabbit IgG (1:500 in PBS, #A11008, Invitrogen).

Overall Aβ immunofluorescence was performed on sagittal brain slices from 15-month-old TgF344-AD and WT rats. Sections were also blocked with Mouse on Mouse (M.O.M.) immunodetection kit (Vector Laboratories) according to kit instructions before permeabilization. The primary antibody was mAb3D6 (murine version of Bapi [47]) diluted to 4 µg/ml in M.O.M. diluent. The secondary antibody was Alexa Fluor 488 goat-anti-mouse IgG (1:500 in PBS, #A11029, Invitrogen).

#### Biochemical Aβ analysis

Brain Aβ aggregate concentrations in rats that underwent PET scanning were measured with sandwich ELISA as previously described [28]. Briefly, isolated cortex, hippocampus, and cerebellum were homogenized separately with 4 × 10 s spins at 5500 rpm in a Precellys® Evolution (Bertin Instruments, Montigny-le-Bretonneux, France) at a 1:5 weight-to-volume ratio in Tris-buffered saline (TBS) with Complete Protease Inhibitor Cocktail Tablets (Roche Diagnostics International AG, Rotkreuz, Switzerland). Samples were centrifuged at 16,000 *g* at 4 °C for 1 h. Supernatants were collected carefully. Pellets were homogenized in 70% FA at a weight-to-volume ratio of 1:5 and centrifuged at 16,000 *g* at 4 °C for 1 h. Again, supernatants were collected.

The concentration of soluble Aβ aggregates from each brain region was measured by coating a 96-well half-area plate with 145 ng/well of mAb3D6 overnight and blocking with 1% BSA in PBS for 1 h. Aβ protofibrils (BioArctic) were used as standard. TBS brain extracts were diluted 1:200 and incubated overnight at 4 °C, then detected with biotinylated 3D6 (0.5 µg/ml) and streptavidin-HRP (1:3000, Mabtech AB, Nacka Strand, Sweden). Signals were developed with K blue aqueous TMB substrate, stopped with 1 M H_2_SO_4_ and read with a spectrophotometer at 450 nm. In GraphPad Prism 9.1.0, a sigmoidal, 4PL (X is concentration) standard curve was plotted for the interpolation of sample concentrations. All dilutions were made in ELISA incubation buffer.

For total Aβ_40_ and Aβ_42_ concentrations, 96-well half-area plates were coated with 100 ng of polyclonal rabbit anti-human Aβ_40_ (custom production from Agrisera, Vännäs, Sweden) or monoclonal rabbit anti-human Aβ_42_ (#700,254, Invitrogen), respectively. The next day, plates were blocked with 1% BSA in PBS for 1 h. Aβ_40_ (Innovagen) and Aβ_42_ (Innovagen) were used as standards respectively. FA brain extracts were neutralized with 2 M Tris, diluted 1:10,000 and incubated overnight at 4 °C. The procedure continued as described above for the soluble Aβ aggregate ELISA.

### Statistical analyses

Statistical analyses were performed in GraphPad Prism 9.1.0 (GraphPad Software, Inc., San Diego, CA). Results are reported as mean ± standard deviation. Statistical assessment was carried out by two-tailed *t*-test, one-way ANOVA with Tukey’s multiple comparisons test or two-way ANOVA with Šídák’s multiple comparisons test. Linear correlation was expressed by Pearson’s correlation coefficient.

## Results

**Fig. 1:**
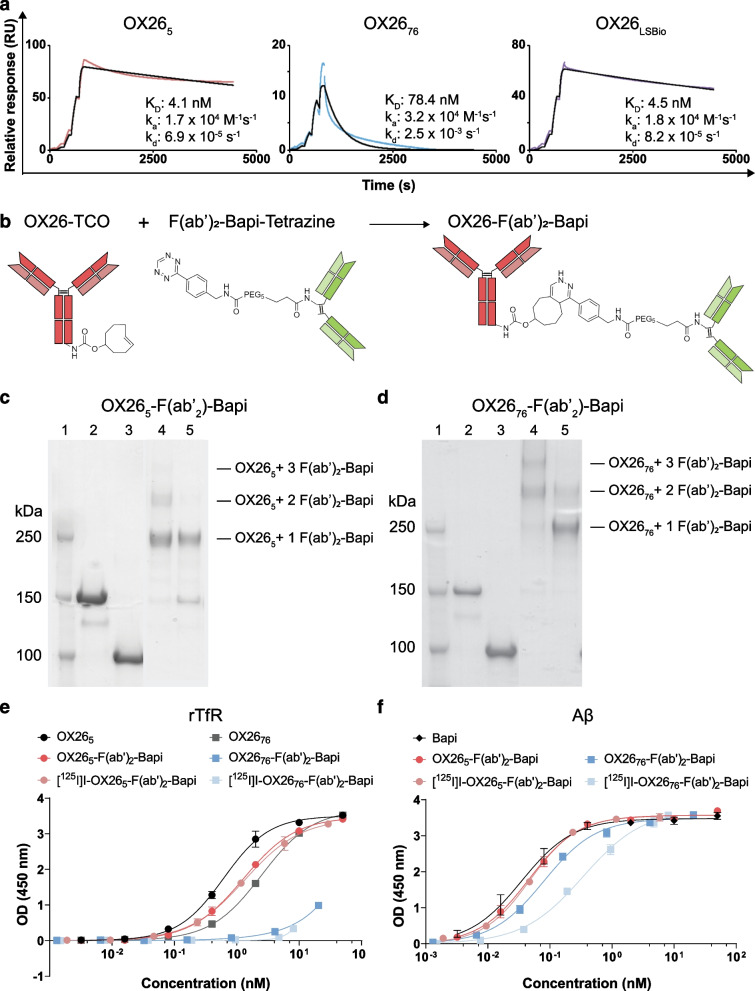
Generation of bispecific fusion proteins with OX26 affinity variants. **a** Biacore analysis of affinity to rTfR for OX26_5_, OX26_76_ and commercial OX26_LSBio_. **b** Schematic of the IEDDA reaction to generate OX26-F(ab′)_2_-Bapi bispecific fusion proteins. **c, d** SDS-PAGE analysis of OX26_5_-F(ab′)_2_-Bapi (**c**) and OX26_76_-F(ab′)_2_-Bapi (**d**). Lanes 1, molecular weight ladder; 2, parental OX26 IgG; 3, F(ab′)_2_-Bapi; 4–5, preparative SEC fractions for the final pool of bispecific fusion protein. **e, f** ELISA analysis of OX26_5_-F(ab′)_2_-Bapi and OX26_76_-F(ab′)_2_-Bapi before and after ^125^I-labelling binding to rTfR (**e**) and Aβ (**f**). RU, response unit; OD, optical density

The *K*_D_ of OX26_5_ was 4.1 nM and the *K*_D_ of OX26_76_ was 78.4 nM (Fig. 1a), which were consistent with literature [22]. The *K*_D_ of the OX26_5_ variant was also similar to that of the commercial control, OX26_LSBio_. Immunofluorescent staining with mAb3D6, the murine version of Bapi, illustrated that this antibody can bind to the Aβ pathology in TgF344-AD rats but not in WT tissues (Additional file 1: Fig. S1). Therefore, Bapi was a suitable anti-Aβ antibody for use in the bispecific fusion proteins. OX26_5_ or OX26_76_ was chemically conjugated to F(ab′)_2_-Bapi to produce two bispecific fusion proteins: OX26_5_-F(ab′)_2_-Bapi and OX26_76_-F(ab′)_2_-Bapi (Fig. 1b). The final bispecific fusion protein products contained OX26_5_ or OX26_76_ conjugated to 1–3 F(ab′)_2_-Bapi, as identified by SDS-PAGE analysis (Fig. 1c, d). Compared to OX26_5_, OX26_5_-F(ab′)_2_-Bapi retained its binding to rTfR before and after ^125^I-labelling (Fig. 1e; Table 3). Conversely, OX26_76_-F(ab′)_2_-Bapi lost most of its binding to rTfR compared with OX26_76_. The EC50 of OX26_76_-F(ab′)_2_-Bapi, both before and after ^124^I-labelling, was unmeasurable in the given ELISA assay. Both bispecific fusion proteins retained their binding to Aβ before and after radiolabelling compared to the full Bapi IgG (Fig. 1f; Table 3).

**Table 3:** EC50 of antibody binding to rTfR or Aβ in direct ELISA

ELISA target	OX26_5_	OX26_76_	Bapi	OX26_5_-F(ab′)_2_-Bapi		OX26_76_-F(ab′)_2_-Bapi	
				Unlabelled	[^125^I]-labelled	Unlabelled	[^125^I]-labelled
rTfR	0.62	2.54	–	1.28	1.36	Undetectable	Undetectable
Aβ	–	–	0.03	0.05	0.05	0.08	0.34

### [^125^I]I-OX26_5_-F(ab′)_2_-Bapi had higher brain uptake than [^124^I]I-OX26_76_-F(ab′)_2_-Bapi in WT rats

**Fig. 2:**
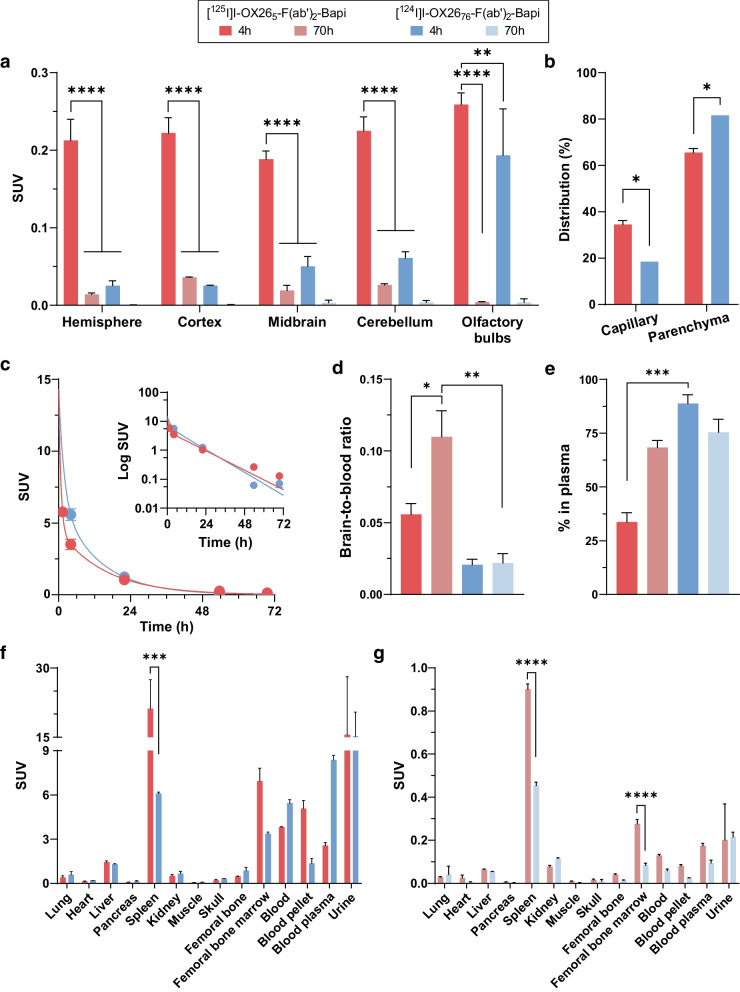
Ex vivo biodistribution of [^125^I]I-OX26_5_-F(ab′)_2_-Bapi or [^124^I]I-OX26_76_-F(ab′)_2_-Bapi in WT rats. **a** Concentration (SUV) of [^125^I]I-OX26_5_-F(ab′)_2_-Bapi or [^124^I]I-OX26_76_-F(ab′)_2_-Bapi in WT rat brain regions 4 h and 70 h post-administration. **b** Bispecific fusion protein distribution in brain capillaries and parenchyma 4 h post-administration. **c** Whole blood elimination curves over 3 days post-administration. Curve fit based on a two-phase decay non-linear regression model. **d, e** Brain-to-blood ratio (**d**) and percent in plasma (**e**) 4 h and 70 h post-administration. **f, g** Peripheral ex vivo biodistribution (SUV) of [^125^I]I-OX26_5_-F(ab′)_2_-Bapi or [^124^I]I-OX26_76_-F(ab′)_2_-Bapi in WT rats 4 h (**f**) and 70 h (**g**) post-administration. *P ≤ 0.05, **P ≤ 0.01, ***P ≤ 0.001, ****P ≤ 0.0001

Four hours post-injection, the concentration of [^125^I]I-OX26_5_-F(ab′)_2_-Bapi in WT rats was 1.3- to 9-fold higher than the concentration of [^124^I]I-OX26_76_-F(ab′)_2_-Bapi in the different brain regions measured (*P* < 0.01, Fig. 2a). Capillary depletion 4 h post-injection determined that the majority of the bispecific fusion protein was distributed to the parenchyma (Fig. 2b), with a slightly higher parenchymal portion for [^124^I]I-OX26_76_-F(ab′)_2_-Bapi (*P* = 0.03). The whole blood exposure, measured as the AUC, was similar between the bispecific fusion proteins (Fig. 2c), although [^125^I]I-OX26_5_-F(ab′)_2_-Bapi had faster elimination than [^124^I]I-OX26_76_-F(ab′)_2_-Bapi in the fast distribution phase (Table 4). The brain-to-blood ratio for [^125^I]I-OX26_5_-F(ab′)_2_-Bapi was significantly higher 70 h post-injection compared with 4 h post-injection (*P* = 0.024) while there was no difference between the two time points for [^124^I]I-OX26_76_-F(ab′)_2_-Bapi (*P* = 0.999; Fig. 2d). The brain-to-blood ratio 70 h post-administration was also significantly higher for [^125^I]I-OX26_5_-F(ab′)_2_-Bapi compared to [^124^I]I-OX26_76_-F(ab′)_2_-Bapi (*P* = 0.004), indicating that [^125^I]I-OX26_5_-F(ab′)_2_-Bapi entered the brain to a higher extent. The percent in plasma, indicative of the amount of free ligand in the blood, was significantly higher for [^124^I]I-OX26_76_-F(ab′)_2_-Bapi compared to [^125^I]I-OX26_5_-F(ab′)_2_-Bapi at 4 h post-administration (*P* = 0.018, Fig. 2e). The peripheral biodistribution was similar for both fusion proteins at 4 h and 70 h post-administration. There was significantly more [^125^I]I-OX26_5_-F(ab′)_2_-Bapi than [^124^I]I-OX26_76_-F(ab′)_2_-Bapi in the spleen at both time points (3.5-fold, *P* = 0.0002 at 4 h and 2-fold, *P* < 0.0001 at 70 h post-injection) and in the bone marrow at 70 h post-injection (3.3-fold, *P* < 0.0001) (Fig. 2f, g). Overall, the ex vivo studies showed that OX26_5_-F(ab′)_2_-Bapi was a more favourable PET ligand candidate .

**Table 4:** Whole blood half-lives and area under the curve (AUC) for bispecific fusion protein variants

Variant	Half-life (h)		AUC
	Fast phase	Slow phase	
[^125^I]I-OX26_5_-F(ab′)_2_-Bapi	0.50 (0.41–0.59)	10.78 (8.02–14.15)	77.34 (69.85–84.84)
[^124^I]I-OX26_76_-F(ab′)_2_-Bapi	1.22 (0–2.41)	9.17 (7.13–25.96)	82.85 (73.09–92.62)

Data presented as mean (95% confidence intervals)

### [^124^I]I-OX26_5_-F(ab′)_2_-Bapi PET visualized Aβ pathology in TgF344-AD rats and correlated with ex vivo brain distribution of [^124^I]I-OX26_5_-F(ab′)_2_-Bapi

**Fig. 3:**
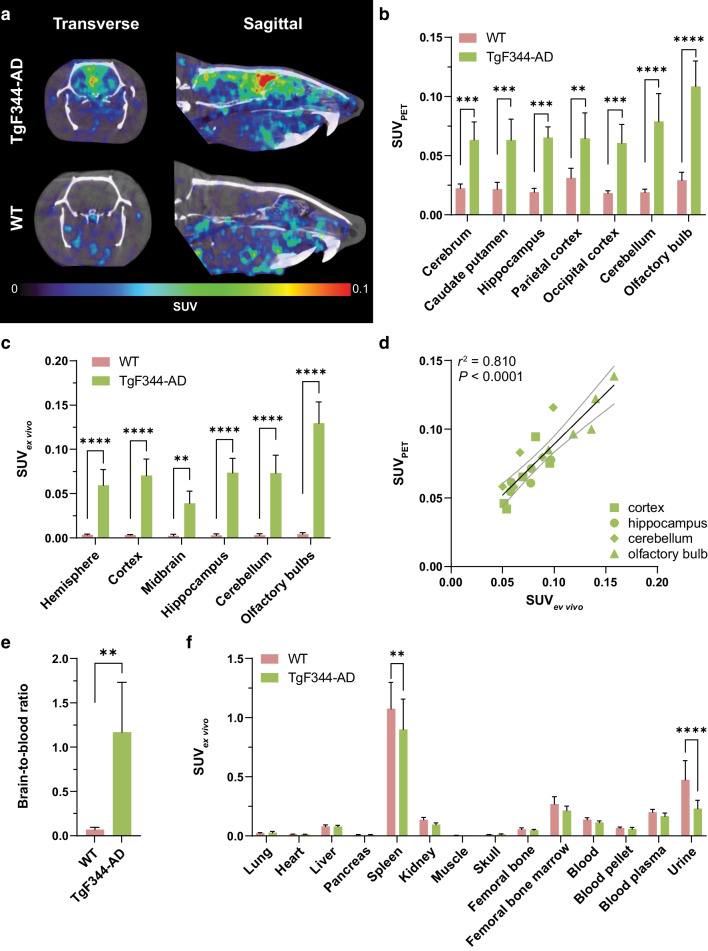
[^124^I]I-OX26_5_-F(ab′)_2_-Bapi PET images and quantification. **a** Representative in vivo PET images from TgF344-AD and WT rats 3 days post-administration of [^124^I]I-OX26_5_-F(ab′)_2_-Bapi. **b** PET image-based quantification of [^124^I]I-OX26_5_-F(ab′)_2_-Bapi distribution (SUV) in WT and TgF344-AD rat brains. **c** Ex vivo [^124^I]I-OX26_5_-F(ab′)_2_-Bapi retention (SUV) in WT and TgF344-AD rat brains. **d** Pearson’s correlation between PET-based quantification (SUV_PET_) and ex vivo retention (SUV_ex vivo_) of [^124^I]I-OX26_5_-F(ab′)_2_-Bapi in four brain regions from TgF344-AD rats. **e** Brain-to-blood ratio in TgF344-AD and WT rats. **f** Peripheral ex vivo biodistribution (SUV) of [^124^I]I-OX26_5_-F(ab′)_2_-Bapi in TgF344-AD and WT rats 3 days post-administration. **P ≤ 0.01, ***P ≤ 0.001, ****P ≤ 0.0001

PET scans of TgF344-AD rats showed increased signal in the brain compared with baseline signals in the brains of WT rats, specifically in the cortex and cerebellum (Fig. 3a). Quantification of PET confirmed that TgF344-AD rats had 2- to 4-fold higher radioligand retention than WT rats, depending on the brain region (*P* < 0.01, Fig. 3b). Furthermore, ex vivo analysis on perfused brains showed significantly higher [^124^I]I-OX26_5_-F(ab′)_2_-Bapi retention in all brain regions in TgF344-AD rats compared with WT rats (18- to 32-fold, *P* < 0.01, Fig. 3c). The PET signal quantification significantly correlated with ex vivo [^124^I]I-OX26_5_-F(ab′)_2_-Bapi concentrations in TgF344-AD rat brains (*r*^2^ = 0.810, *P* < 0.0001, Fig. 3d). The brain-to-blood ratio was also significantly higher in TgF344-AD rats than WT rats (*P* = 0.0003, Fig. 3e). The peripheral distribution of [^124^I]I-OX26_5_-F(ab′)_2_-Bapi was similar between TgF344-AD and WT rats (Fig. 3f), except that the WT animals had higher radioactivity concentrations in the spleen (1.2-fold, *P* = 0.0095) and urine (2.1-fold, *P* < 0.0001) compared with TgF344-AD rats. Finally, the blood pharmacokinetic profile was not affected by genotype. The TgF344-AD and WT rats had similar [^124^I]I-OX26_5_-F(ab′)_2_-Bapi elimination curves in whole blood over 72 h post-administration (Additional file 1: Fig. S2a) and the percent in plasma at 72 h post-administration did not differ significantly (*P* = 0.76, Additional file 1: Fig. S2b).

### [^124^I]I-OX26_5_-F(ab′)_2_-Bapi PET correlated with Aβ pathology

**Fig. 4:**
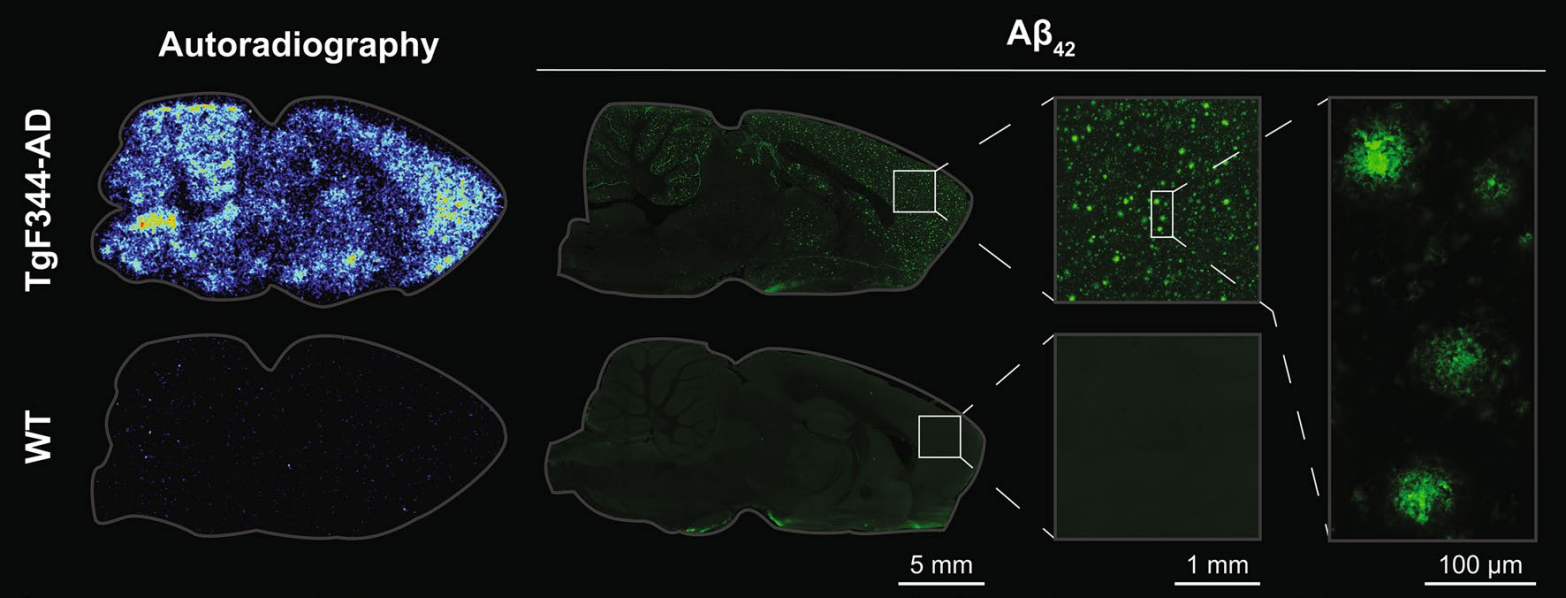
Representative sagittal images of ex vivo autoradiography and Aβ_42_ immunofluorescence from TgF344-AD and WT rats after [^124^I]I-OX26_5_-F(ab′)_2_-Bapi PET

Ex vivo autoradiography performed on perfused brain sections showed increased signals in the cortex and cerebellum of TgF344-AD rats (Fig. 4). Sagittal sections stained for Aβ_42_ illustrated that TgF344-AD rats had abundant Aβ_42_ pathology in the cortex, caudate putamen, hippocampus and cerebellum, displaying both dense and diffuse Aβ deposits (Fig. 4). On the contrary, there was no Aβ_42_ pathology in WT rat brains.

**Fig. 5:**
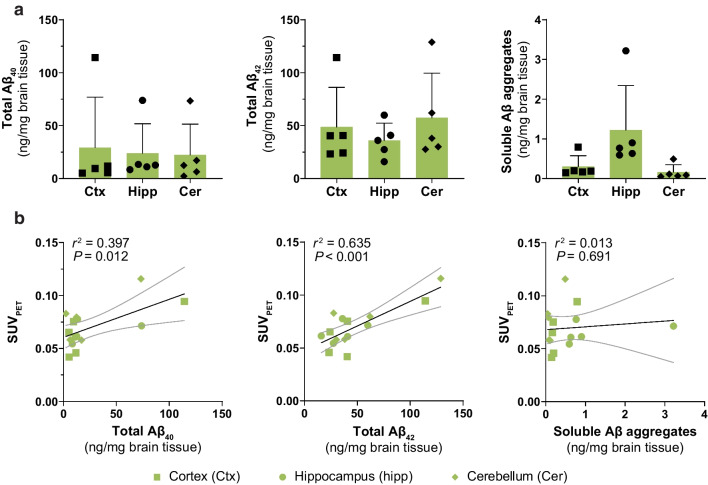
**a** ELISA quantification of total Aβ_40_, total Aβ_42_ and soluble Aβ aggregates in brain regions from TgF344-AD rats. **b** Pearson’s correlation between PET-based quantification (SUV_PET_) and Aβ concentrations

ELISA analysis of brain homogenates revealed that Aβ levels did not differ significantly among cortex, hippocampus and cerebellum in TgF344-AD rats for total Aβ_40_, total Aβ_42_ or soluble Aβ aggregates (Fig. 5a). The PET signal quantification correlated with concentrations of total Aβ_40_ (*P* < 0.05) and Aβ_42_ (*P* < 0.001) but not with soluble Aβ aggregates (Fig. 5b).

## Discussion

To investigate the role TfR affinity plays in transport of bispecific immunoPET ligands across the BBB, two bispecific fusion protein variants were produced. In vivo pharmacokinetic studies indicated that 72 h was a good time point for PET imaging since there was little immunoPET ligand signal left in the brains or blood of WT rats, suggesting good contrast for PET imaging. This time corresponded with immunoPET imaging of Aβ in two different transgenic AD mouse models [7, 28]. Furthermore, we found that at 4 h post-administration, there was significantly more [^125^I]I-OX26_5_-F(ab′)_2_-Bapi in all brain areas compared with [^124^I]I-OX26_76_-F(ab′)_2_-Bapi. These results contradict previous findings that the OX26_76_ IgG enters the brain significantly better than the OX26_5_ IgG [22, 36]. One explanation for the higher BBB transcytosis of [^125^I]I-OX26_5_-F(ab′)_2_-Bapi is that the conjugation reaction may have an effect on the affinity of the parent antibody to rTfR. After conjugation, OX26_5_-F(ab′)_2_-Bapi retained a similar rTfR affinity as the parental OX26_5_ IgG. Unexpectedly, the in vitro affinity of OX26_76_ to rTfR substantially decreased after conjugation, which likely led to the lower brain uptake of [^124^I]I-OX26_76_-F(ab′)_2_-Bapi due to the poor interaction with endogenous TfR [18, 22, 35, 36]. This hypothesis is supported by the higher percent in plasma at 4 h, the longer half-life in the fast distribution phase in whole blood and the lower concentration in the spleen compared with [^125^I]I-OX26_5_-F(ab′)_2_-Bapi, all of which indicate insufficient binding to TfR in vivo.

Dosage has also been suggested to influence the effect of TfR affinity on BBB transcytosis, such that higher-affinity binders enter the brain better at tracer doses [9, 16]. This dose-dependency hypothesis could explain the better brain uptake of [^125^I]I-OX26_5_-F(ab′)_2_-Bapi since the max dose used here was 0.12 mg/kg, while previous reports with these OX26 variants used therapeutic doses of 10–20 mg/kg [22, 36]. Further studies are necessary to determine the extent to which dosing influences the effects of affinity, specifically for bispecific immunoPET radioligands.

ImmunoPET ligands with TfR-mediated brain uptake have proven more sensitive in detecting Aβ pathology than small-molecule tracers in mice [7, 28, 31, 32]. Previous PET analyses of the TgF344-AD rat model with the amyloid ligands [^18^F]FDDNP in 15-month-old rats and [^18^F]Florbetaben in 18-month-old rats both measured small differences in brain pathology between AD and WT rats (1.07- and 1.25-fold increases in signal, respectively) [41, 42]. ImmunoPET with [^124^I]I-OX26_5_-F(ab′)_2_-Bapi in slightly younger TgF344-AD rats (14-month-old) showed 2- to 4-fold higher signals than WT littermates, depending on brain region. Taken together, the data presented here indicate that immunoPET ligands may be more sensitive than small-molecule tracers in rats as well.

In TgF344-AD animals, the brain-region SUVs also correlated with the ex vivo levels of [^124^I]I-OX26_5_-F(ab′)_2_-Bapi and with the total Aβ_40_ and Aβ_42_ levels detected by ELISA. Furthermore, the increased cortical and cerebellar PET signal corresponded with increased ex vivo autoradiography signal and abundant Aβ_42_ staining in these brain regions in TgF344-AD rats. These results suggest that the PET signal originated from radiolabelled bispecific fusion protein specifically binding to and around Aβ deposits in TgF344-AD rat brains. The high cortical, hippocampal and dorsal striatal (caudate and putamen) signals seen in the immunoPET scans, autoradiography and Aβ_42_ immunofluorescence correspond with descriptions of pathology in literature [41–43]. Cerebellar pathology has been reported to develop later than cortical and hippocampal pathology in TgF344-AD rats [41, 43]. Here, we noticed a strong PET signal in the cerebellum, which corresponds with the high concentration of Aβ in the brain tissue. One explanation for this strong cerebellar PET signal in comparison to previous PET studies [42] could be that, due to the overexpression of Aβ_42_ in this model and later development of cerebellar pathology, the cerebellum may contain proportionally more diffuse Aβ plaques than other brain regions. Diffuse deposits represent an earlier form of Aβ plaques [48] that are readily detected with [^124^I]I-OX26_5_-F(ab′)_2_-Bapi but lack the amyloid core that is detected by traditional Aβ tracers.

Another difference from previous findings [28, 30, 32] is that in this study the PET signals did not correlate with the levels of soluble Aβ aggregates within AD animal groups. Furthermore, the correlations with total Aβ_40_ and Aβ_42_ were driven by one AD rat that had higher pathology than the others. This ligand clearly differentiated between AD and WT rats but the lack of strong correlations to Aβ pathology may be because the animals were at the same disease stage. In future studies, the ability of this radioligand to detect pathology changes at different ages and after therapeutic interventions should be explored.

One limitation in this study was the production of bispecific fusion proteins with chemical conjugation. The IEDDA reaction resulted in a heterogeneous product with OX26 IgG conjugated to 1–3 F(ab′)_2_-Bapi fragments. Further, the two moieties (OX26 IgG and F(ab′)_2_-Bapi) were conjugated randomly at different sites. This method is unsuitable to produce a clinically applicable bispecific immunoPET radioligand. An alternative would be to use a site-specific conjugation method, such as Sortase A catalysed chemo-enzymatic reaction [49].

## Conclusion

In conclusion, we have shown that TfR affinity influences the BBB passage of bispecific immunoPET ligands. However, it remains unclear whether the dose of the bispecific antibody influences the effect of affinity. Finally, we have demonstrated that the TfR-mediated transport of an immunoPET radioligand enables sensitive imaging of brain Aβ pathology in a rat model of AD, expanding its use over mice. Therefore, this strategy for delivery of immunoPET ligands to the CNS could eventually be translated from bench to bedside given the development of suitable human-specific TfR-binders.

## Supplementary Information

**Additional file 1. Fig S1**. Representative sagittal images of Aβ pathology visualized with RmAb3D6, the murine version of Bapi, in 15-month-old TgF344-AD and WT rats. **Fig S2**. Ex vivo blood pharmacokinetics of [^124^I]I-OX26_5_-F(ab′)_2_-Bapi in TgF344-AD and WT rats

## Data Availability

The datasets generated and/or analysed during the current study are available from the corresponding author on reasonable request.

## Abbreviations

[^11^C]PiB: [^11^C]Pittsburgh Compound B
Aβ: Amyloid-beta
AD: Alzheimer’s disease
AUC: Area under the curve
Bapi: Bapineuzumab
BBB: Blood–brain barrier
BSA: Bovine serum albumin
CNS: Central nervous system
CT: Computed tomography
FA: Formic acid
HAc: Acetic acid
HRP: Horseradish peroxidase
IEDDA: Inverse electron demand Diels–Alder
MRI: Magnetic resonance image
OD: Optical density
PET: Positron emission tomography
PK: Pharmacokinetic
RT: Room temperature
rTfR: Rat transferrin receptor
SUV: Standardized uptake value
TCO: Trans-cyclooctene
TfR: Transferrin receptor
