# Supplementary material for: ImmunoPET imaging of amyloid-beta in a rat model of Alzheimer’s disease with a bispecific, brain-penetrating fusion protein
Source: Transl Neurodegener. 2022 Dec 26;11:55. doi: 10.1186/s40035-022-00324-y (PMC9791759; doi:10.1186/s40035-022-00324-y)
Supplement: Supplementary file 1 — Additional file 1. Fig S1. Representative sagittal images of Aβ pathology visualized with RmAb3D6, the murine version of Bapi, in 15-month-old TgF344-AD and WT rats. Fig S2. Ex vivo blood pharmacokinetics of [124I]I-OX265-F(ab′)2-Bapi in TgF344-AD and WT rats [file 40035_2022_324_MOESM1_ESM.docx]

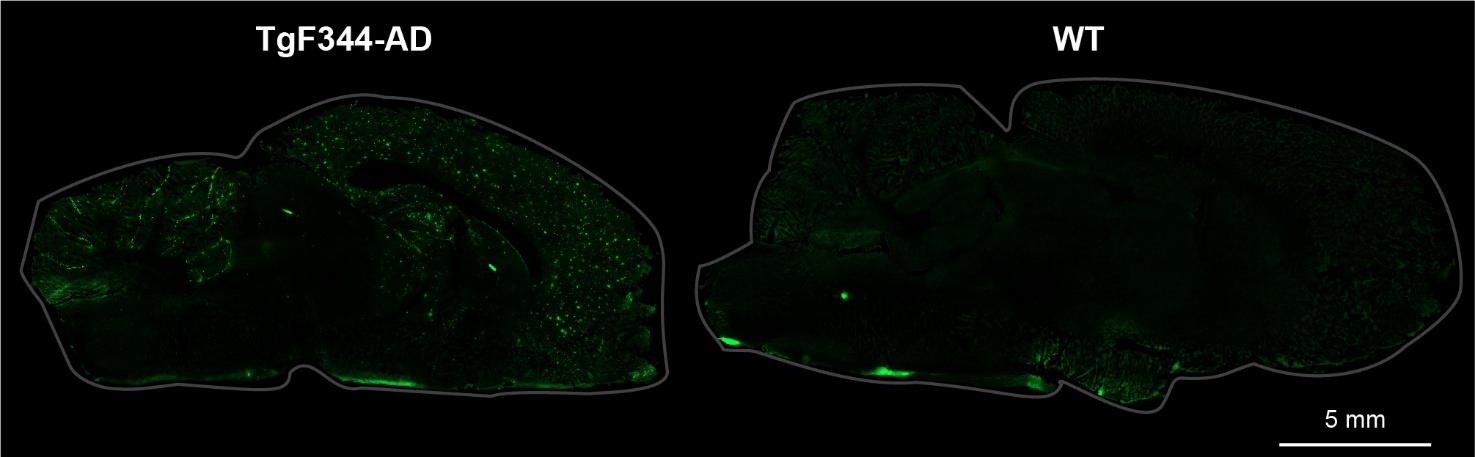


Fig S1. Representative sagittal images of Aβ pathology visualized with RmAb3D6, the murine version of Bapi, in 15 month old TgF344-AD and WT rats.


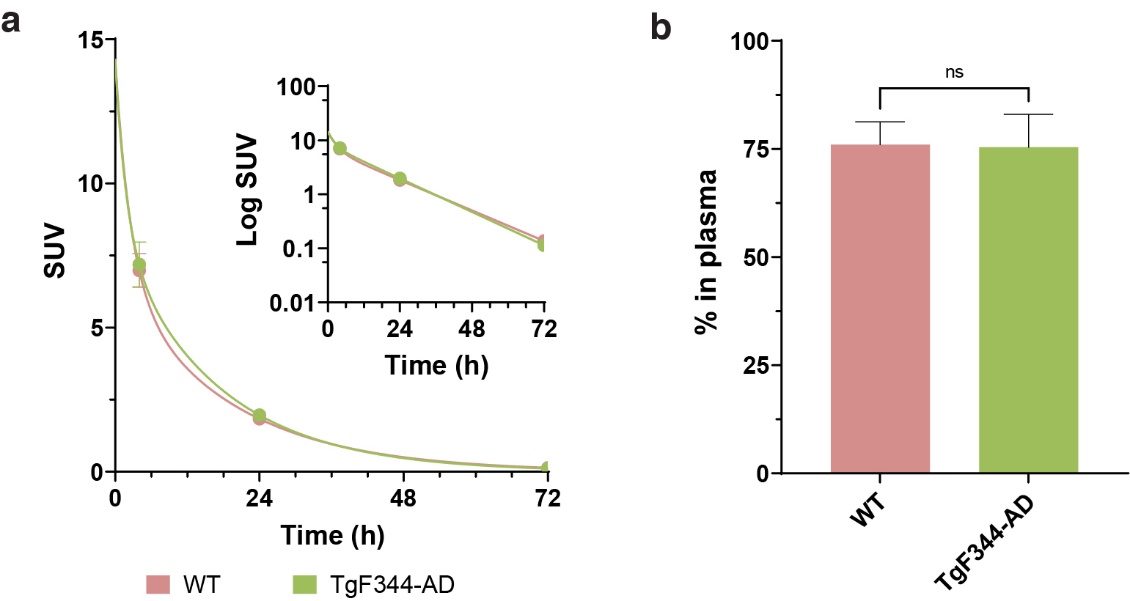


Fig S2. *Ex vivo* blood pharmacokinetics of [^124^I]I-OX26_5_-F(ab’)_2_-Bapi in TgF344-AD and WT rats. (**a**) Whole blood elimination curves over 72 h post-administration. Curve fit based on a two-phase decay non-linear regression model. (**b**) Percent in plasma 72 h post-administration.
